# Hemolytic uremic syndrome related to Shiga-like toxin-producing *Escherichia coli* with encephalitis hiding a human herpesvirus-6 infection: a case report

**DOI:** 10.1186/s13256-021-02873-8

**Published:** 2021-05-25

**Authors:** Sophie Mounier, Arthur Gavotto, Julie Tenenbaum, Pierre Meyer, Marc Fila, Julien Baleine

**Affiliations:** 1 Department of Neonatal Medicine and Pediatric Intensive Care, Arnaud de Villeneuve Hospital, Montpellier University Hospital Center, 371 Avenue du Doyen Gaston Giraud, 34295 Montpellier Cedex 5, France; 2 Department of Pediatric Cardiology, Arnaud de Villeneuve Hospital, Montpellier University Hospital Center, 371 Avenue du Doyen Gaston Giraud, 34295 Montpellier Cedex 5, France; 3 CNRS, INSERM, University of Montpellier, PhyMedExpMontpellier, France; 4 Department of Pediatric Nephrology, Arnaud de Villeneuve Hospital, Montpellier University Hospital Center, 371 Avenue du Doyen Gaston Giraud, 34295 Montpellier Cedex 5, France; 5 Department of Pediatric Neurology, Gui de Chauliac Hospital, Montpellier University Hospital Center, 80 Avenue Augustin Fliche, 34090 Montpellier, France

**Keywords:** Hemolytic uremic syndrome, Pericarditis, Human herpesvirus-6

## Abstract

**Background:**

Cardiac and neurological involvement in hemolytic uremic syndrome are life-threatening complications. The most frequent complications of cardiac involvement in hemolytic uremic syndrome are myocarditis and cardiac dysfunction due to fluid overload. Pericarditis remains very rare in hemolytic uremic syndrome. To our knowledge, only five cases of cardiac tamponade associated with hemolytic uremic syndrome have been described in literature.

**Case summary:**

A 27-month-old Caucasian girl presented with symptoms of nonbloody diarrhea and tonic-clonic seizures. The diagnosis of Shiga-like toxin-producing *Escherichia coli* hemolytic uremic syndrome with central nervous system involvement was made, and stool examination revealed infection with a Shiga-like toxin-producing *Escherichia coli*. She did not need renal replacement therapy but had severe neurological impairment. The patient’s course was complicated by pericardial effusion. A pericardiocentesis was performed via an apical approach because the pericardial effusion was predominantly surrounding the left ventricle. Effusion analysis showed an exudate and positivity for human herpesvirus-6B on polymerase chain reaction with viremia. This finding was consistent with primary human herpesvirus-6 infection with encephalitis.

**Conclusion:**

We report this uncommon case of Shiga-like toxin-producing *Escherichia coli* hemolytic uremic syndrome associated with a severe human herpesvirus-6 infection. Secondary isolated pericardial effusion and atypical neurological involvement are uncommon in Shiga-like toxin-producing *Escherichia coli* hemolytic uremic syndrome and should lead the physician to perform additional investigations.

## Background

Hemolytic uremic syndrome (HUS) is defined by microangiopathic hemolytic anemia with thrombocytopenia and acute kidney injury [1]. The most common cause of pediatric HUS is Shiga-like toxin-producing *Escherichia coli* (STEC), accounting for 90% of cases [2]. Management of STEC HUS is primarily based on supportive care [3]. Extrarenal manifestations can occur and affect other organ systems, including central nervous system, heart, pancreas, liver, and gastrointestinal tract [4]. Severe central nervous system involvement leads to poor prognosis. Treatment with eculizumab, a monoclonal antibody against complement factor C5, that blocks complement activation, appears to improve neurologic outcomes [5].

Pericarditis during HUS is a rare complication. Cardiac complications occur in approximately 5–10% of HUS cases. Cardiac dysfunction or direct cardiac involvement related to myocarditis, thrombotic microangiopathy, and tamponade, with pericardial blood leading to tamponade, are the most frequent manifestations, whereas pericarditis is rare [3, 6]. To detect early cardiac involvement, according to the French recommendations, troponin levels and cardiac function are monitored in STEC HUS [4]. To date, only five cases of cardiac tamponade have been described in literature [7–11]. Human herpesvirus-6 (HHV-6) infection combined with STEC HUS has not been reported. We present a case of pericardial effusion requiring pericardiocentesis in a patient with HUS-associated HHV-6 infection with encephalitis.

## Case presentation

A 27-month-old Caucasian girl without relevant medical history presented with nonbloody diarrhea with moderate volume depletion. There was no vomiting. Supportive treatment with oral rehydration solution was given. She had exhibited a cutaneous rash 3 days before clinical manifestations but no fever. The next day, she developed tonic-clonic seizures that were treated with clonazepam in a nontertiary pediatric emergency department.

The first biological exams showed acute kidney injury (serum creatinine level 131 µmol/L, urea 34.2 mmol/L) without disturbance of blood electrolytes (sodium 141 mmol/L, potassium 4 mmol/L), with normal diuresis. There were signs of hemolytic anemia (9.9 g/dL), an increased LDH level (1470 U/L), a haptoglobin level < 0.1 g/L, and 2.6% schistocytes, and a low platelet count (46 G/L).

She was immediately referred to the pediatric intensive care unit (PICU) because of severe central nervous system involvement. Enterohemorrhagic *E. coli* producing Shiga-like toxin 2 genes O80 serotype was found in stool sample. The diagnosis of hemolytic uremic syndrome due to Shiga toxin-producing *E. coli* was made.

During disease evolution, she had diuresis and did not need renal replacement therapy. She had persistent diarrhea for two weeks. Abdominal ultrasound showed a normal renal resistance index with colitis and ascites. Liver function tests stayed in the normal ranges. After 2 weeks, she had normal renal function with persistent proteinuria.

Regarding her neurologic outcome, upon admission to the PICU, she was drowsy, but responsive to sound stimuli, had lower limb hypertonia and increased deep tendon reflexes. Eculizumab was administered according to French Pediatric Nephrology Society guidelines because of severe associated central nervous system involvement. She was administered the vaccine against serogroups A, C, B, W-135, Y of meningococcus, and given prophylactic oracillin. She was initially treated with clonazepam, fosphenytoin, and phenobarbital. She went into status epilepticus on day 3 and needed additional treatment with a loading dose of levetiracetam. She did not have additional seizures and was gradually weaned from the antiepileptic drugs, with only levetiracetam remaining on day 10. Cerebral magnetic resonance imaging (MRI) showed bilateral white-matter hyperintensities affecting the frontal and parietal lobes compatible with neurological involvement in HUS. Electroencephalography (EEG) patterns suggested encephalitis. No cerebrospinal fluid (CSF) analysis was performed because of the initial diagnosis of central nervous system involvement in HUS. Tetraparesis, severe cognitive impairment with loss of speech, difficulty swallowing, and deficits in motor coordination developed. After 1 weeks, a second cerebral MRI showed widespread T2 hyperintensities affecting the external capsules, lenticular nucleus, and thalamus with brain atrophy. After one year, she had persistent neurological impairment with loss of speech and severe psychomotor retardation.

**Fig. 1.**
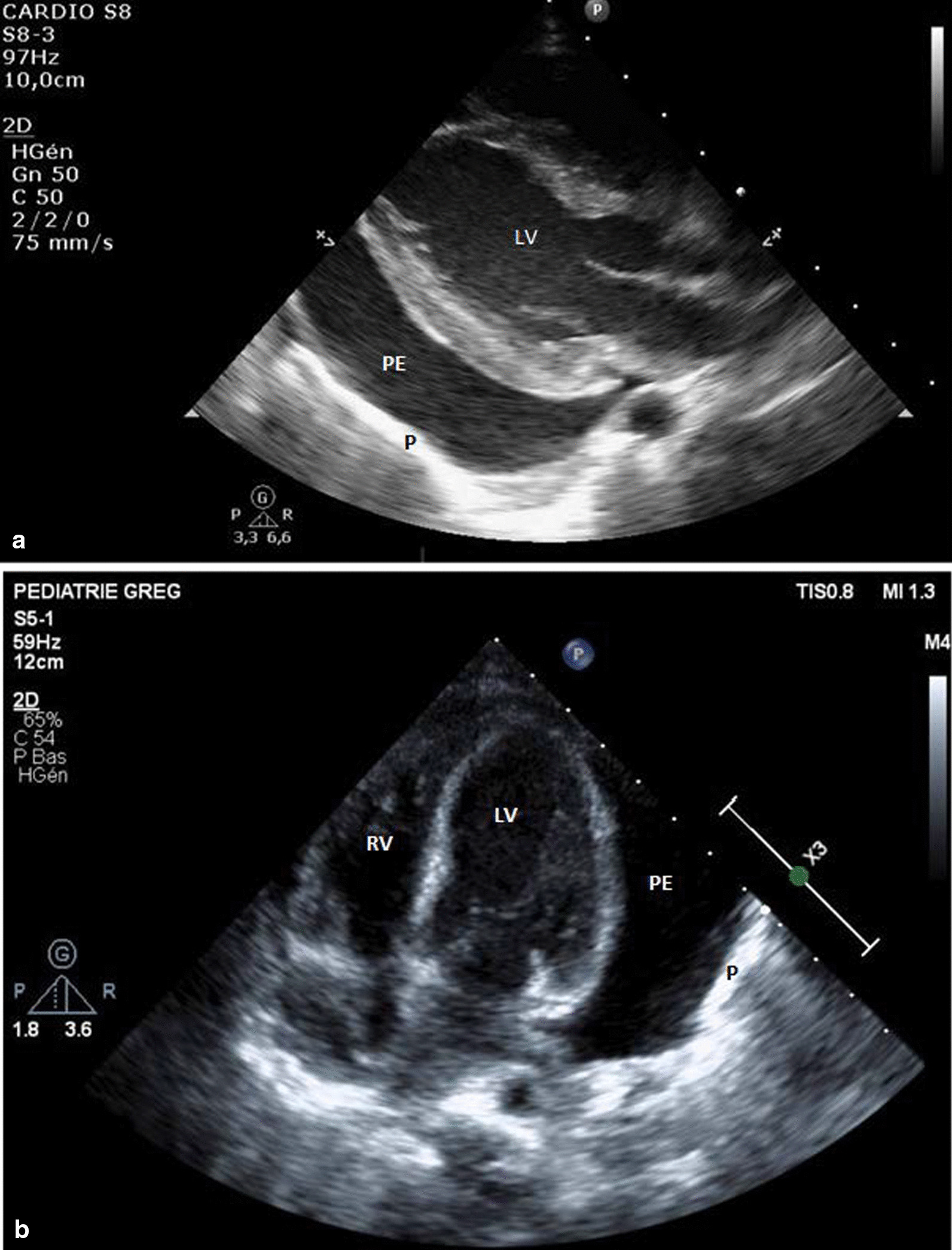
Echocardiography showing pericardial effusion predominantly surrounding the left ventricle before pericardiocentesis. **a** Parasternal long-axis view. **b** Apical four-chamber view. *P *pericardium, *PE *pericardial effusion, *RV *right ventricle, *LV *left ventricle

With regard to the hemodynamic features, the initial evaluation showed that her troponin-T level was 17 ng/ml with normal cardiac function and high blood pressure. The cardiopulmonary clinical examination was normal. She gradually developed tachycardia. On day 5, the daily echocardiography showed circumferential pericardial effusion predominantly surrounding the left ventricle, without clinical or echocardiographic hemodynamically significant signs. This finding was contrary to the observed improvements in renal function and hemolysis. There was no pleural or peritoneal effusion. Her troponin levels was 172 ng/L. Colchicine was initiated on day 10 but did not affect the effusion volume. Pericardial effusion increased, and signs of cardiac tamponade were observed on day 13, including a swinging motion of the heart within the effusion, variations in transvalvular blood flow velocities during inspiration (the flow velocity integral across the pulmonary valve increased to 33%, that across the tricuspid valve increased to 65%, that across the aortic valve decreased to 18%, and that across the mitral valve decreased to 28%), and a plethoric inferior vena cava with minimal respiratory variation. Electrocardiography (ECG) showed only sinus tachycardia. Pericardial effusion was predominantly around the left ventricle (Fig. 1) and extended less than 1 cm around the right ventricle. Pericardiocentesis was performed on day 14. After failure of the subxiphoid approach due to the characteristics of the pericardial effusion, an apical approach was adopted and pericardial drainage device was installed. No complications occurred, and 160 mL citrine fluid was drained. The effusion was an exudate with 44/mm^3^ cells, LDH level of 518 U/L, and total protein content of 33.4 g/L. HHV-6B qualitative polymerase chain reaction (PCR) was positive without bacterial growth associated with viremia (11,316 cp/ml). The pericardial drain was removed after 3 days with any recurrence of the pericardial effusion.

## Discussion

We report an exceptional case of cardiac tamponade during STEC HUS with severe neurological involvement, associated with a primary HHV-6 infection.

HHV-6 is a member of the *Herpesviridae* family and replicates in activated CD4^+^ lymphocytes. The classic manifestation of primary HHV-6 infection in immunocompetent children is roseola infantum. HHV-6 can lead to severe complications [12, 13] such as hepatitis [14], myocarditis [15], and encephalitis [12]. However, it is difficult to establish HHV-6 as the causative agent in severe infections. Furthermore, chromosomally integrated HHV-6 is present in 1% of the population [16] and can lead to an incorrect diagnosis of HHV-6-associated viremia or encephalitis. In our case, chromosomal integration of HHV-6 was ruled out by negative blood PCR for HHV-6 two months later.

During HUS, neurological manifestations are the results of the combined effects of Shiga toxin-induced vascular injury, disordered electrolyte, hypertension, and endothelial dysfunction. Neurological involvement is correlated with the severity of renal impairment, which usually requires temporary renal replacement therapy, and the severity of hemolysis [20]. In our case, severe neurological involvement was not correlated with the severity of HUS and could have been related to the HHV-6 infection.

Drowsiness and inability to communicate are symptoms of HHV-6-associated encephalitis [12]. However, an active HHV-6 infection, indicated by viremia, may not indicate an active infection in the central nervous system [17]. The gold-standard method of identifying HHV-6 encephalitis is quantitative PCR in cerebrospinal fluid [18], which was not performed in our case because of the primary hypothesis of central nervous system involvement in HUS. Clinical features compatible with severe primary HHV-6 infection included a rash 3 days before the clinical manifestations of HUS, dynamic viral replication in the blood, age less than 3 years, and compatible neurological clinical symptoms and neuroimaging characteristics [19]. HHV-6 infection was likely the main cause of her neurological impairment and the poor outcome.

In addition, rare cases of pericarditis in STEC HUS have been described. Cardiac complications are rare, and the most common is myocarditis [3, 6]. Rigamonti et al. [6] performed a review of the literature in 2016 and identified 18 cases involving cardiac complications ≤ 25 days after HUS was diagnosed. The causes were microangiopathy, pericardial blood causing tamponade, and myocarditis. In total, 37% of the patients died and 2 patients experienced cardiac tamponade. Four case reports of cardiac tamponade have been described, and all patients needed pericardiocentesis [7–10]. One additional case report described suspected fatal cardiac tamponade in a patient with HUS [11]. The pericardial effusions analyzed were always exudative and not related to fluid overload. Water-sodium depletion would not be effective for the treatment of pericardial effusion, and pericardiocentesis is likely the appropriate treatment for persistent and significant pericardial effusion in patient with HUS. In our case, the pericardial effusion was secondary and possibly related to the HHV-6 infection as indicated by its late onset during the period of improvement in HUS, which led us to diagnose the HHV-6 infection.

## Conclusion

We report an uncommon case of STEC HUS concurrent with severe HHV-6 infection. Secondary isolated pericardial effusion and neurological involvement are uncommon in STEC HUS and should lead physicians to perform additional investigations.

## Data Availability

Not applicable.
